# The neuronal-specific isoform of BIN1 regulates β-secretase cleavage of APP and Aβ generation in a RIN3-dependent manner

**DOI:** 10.1038/s41598-022-07372-4

**Published:** 2022-03-03

**Authors:** Raja Bhattacharyya, Catarina Amelia Fidalgo Teves, Alexandra Long, Madison Hofert, Rudolph E. Tanzi

**Affiliations:** Genetics and Aging Research Unit, MassGeneral Institute for Neurodegenerative Disease, Henry and Allison McCance Center for Brain Health, Department of Neurology, Massachusetts General Hospital, Harvard Medical School, Boston, MA USA

**Keywords:** Cell biology, Neuroscience

## Abstract

Genome-wide association studies have identified *BIN1 (Bridging integrator 1)* and *RIN3 (Ras and Rab interactor 3)* as genetic risk factors for late-onset Alzheimer’s disease (LOAD). The neuronal isoform of BIN1 (BIN1V1), but not the non-neuronal isoform (BIN1V9), has been shown to regulate tau-pathology and Aβ generation via RAB5-mediated endocytosis in neurons. BIN1 directly interacts with RIN3 to initiate RAB5-mediated endocytosis, which is essential for β-secretase (BACE1)-mediated β-secretase cleavage of β-amyloid precursor protein (APP) to generate Amyloid-β (Aβ), the key component of senile plaques in AD. Understanding the regulatory roles of BIN1 (neuronal BIN1V1) and RIN3 in β-secretase mediated cleavage of APP and Aβ generation is key to developing novel therapeutics to delay or prevent AD progression. Neuronal and non-neuronal isoforms of BIN1 (BIN1V1 and BIN1V9, respectively) were introduced with RIN3 into an in vitro cell-based system to test RIN3-dependent effects of neuronal BIN1V1 and non-neuronal BIN1V9 on β-secretase-mediated cleavage of APP and Aβ generation. Confocal microscopy was performed to examine RIN3-dependent subcellular localization of BIN1V1 and BIN1V9. Western blot analysis was performed to assess the effects of RIN3 and BIN1V1/BIN1V9 on β-secretase mediated processing of APP. We enriched cells expressing BIN1V1 without or with RIN3 via FACS to measure Aβ generation using Aβ ELISA assay, and to evaluate APP internalization by chasing biotinylated or antibody-labeled cell surface APP. Neuronal BIN1V1 containing the CLAP domain and non-neuronal BIN1V9 lacking the CLAP domain are the major isoforms present in the brain. Employing confocal microscopy, we showed that RIN3 differentially regulates the recruitment of both BIN1V1 and BIN1V9 into RAB5-endosomes. We further showed that BIN1V1, but not BIN1V9, downregulates β-secretase (BACE1)-mediated processing of APP in a RIN3-dependent manner. Overexpression of BIN1V1 also attenuated Aβ generation in a RIN3-dependent manner. Using cell-based internalization assays, we show BIN1V1, but not BIN1V9, delays the endocytosis of APP, but not of BACE1, into early endosomes, thereby spatially and temporally separating these two proteins into different cellular compartments, resulting in reduced cleavage of APP by BACE1 and reduced Aβ generation—all in a RIN3-dependent manner. Finally, we show that RIN3 sequesters BIN1V1 in RAB5-positive early endosomes, likely via the CLAP-domain, resulting in attenuated β-secretase processing of APP and Aβ generation by delaying endocytosis of APP. Our findings provide new mechanistic data on how two AD-associated molecules, RIN3 and BIN1 (neuronal BIN1V1), interact to govern Aβ production, implicating these two proteins as potential therapeutic targets for the prevention and treatment of AD.

## Introduction

Amyloid β (Aβ) peptide is derived from the β-amyloid protein precursor (APP) and is the principal component of senile plaque cores in Alzheimer’s disease (AD) brains. Aβ is generated from APP following sequential cleavage by the β-site APP cleaving enzyme (BACE1) and γ-secretase. Processing of APP by BACE1 occurs mainly in RAB5-positive early endosomes. Endocytosis and cell surface trafficking of APP together with BACE1 are critical for Aβ production^1^. Several regulatory proteins are involved in APP endocytosis; most common among them are Dynamin1, Mint1 and 2, and Rab family of small GTPases. Rab GTPase proteins, particularly RAB5, are strongly associated with internalization and endocytosis pathways in AD^2^. Genomic variants in several genes that are involved in membrane trafficking have been linked to increased risk for late-onset AD (LOAD), *e.g. BIN1, PICALM, CD2AP, CD33, EPHA, RIN3, MEF2C, and PTK2B*^3–9^, providing strong evidence for the role of membrane trafficking in AD pathogenesis. *BIN1*, the second-most impactful LOAD susceptibility gene after *APOE,* has been previously implicated in synaptic vesicle endocytosis^10^.

The *BIN1* gene undergoes complex alternate splicing to generate multiple isoforms (BIN1V1- BIN1V10) with diverse tissue and cellular distribution, including in the brain. BIN1 variants BIN1V1, V2 and V3 contain the clathrin and AP-2-binding sites (CLAP domain) at residues 334–376^11^, and are primarily expressed in neurons or astrocytes, while variants BIN1V6, V9, V10 and V12 lacking the CLAP-domain are expressed in oligodendrocytes or microglia^12,13^. Despite the fact that total BIN1 mRNA levels are increased in AD brains^14^, several reports show that the protein level of the neuronal variant (BIN1V1) is decreased, while BIN1V9 is increased in AD^15^. A recent study revealed a nearly 80% reduction in the protein levels of BIN1V1 and ~ sevenfold increase in the levels of BIN1V9 in APP/PS1 transgenic mouse brains^16^. De Rossi et al.^13^ showed that BIN1 is primarily expressed in mature oligodendrocytes showing a significant correlation with the process of postnatal myelination in the brain. This contrasts with the neuronal-specific expression of the BIN1 paralog, Amphiphysin 1. The neuronal isoform BIN1V1 is the largest BIN1 variant, while the non-neuronal isoform BIN1V9 is the smallest BIN1 variant. Neuronal BIN1V1, but not BIN1V9, acts as a negative regulator of RAB5-mediated endocytosis in neurons and has been shown to regulate the propagation of tau-pathology^17^. Meanwhile, the role of either BIN1 variant in APP processing and Aβ generation has remained unclear.

BIN1 and RAB5 interact via the BIN1-binding partner, RIN3^4,18,19^. RIN3 is a guanidine nucleotide exchange factor (GEF), that activates members of the RAB5 family (RAB5, 21, 22, 24 and 31) involved in endocytosis, intracellular vesicular trafficking^18,20,21^. Overexpression studies have shown that RIN3 recruits the BIN1 paralog Amphiphysin II into RAB5-positive endosomes^18^. The increased level of the neuronal isoform of BIN1 (BIN1V1) in AD is speculated to sequester RIN3 to prevent efficient RAB5 activation, while loss/absence of BIN1 results in ‘‘more-free’’ RIN3 that activated RAB5 in neuron^17^. Wild type RIN3 and a newly identified missense RIN3 variant (W63C) may contribute to AD pathogenesis^22^. GWAS studies have identified genome-wide significant associated of AD risk at the SLC24A4/RIN3 locus^23^. More recently, quantitative PCR and protein analysis have shown a significant increase in the mRNA and expression levels of RIN3 in the neurons of APP/PS1 transgenic compared to non-transgenic mice^16^. Increased levels of RIN3 in neurons is accompanied by increased early endosome enlargement, which is a hallmark of early onset AD^24,25^. In vitro studies in PC12 cells have revealed that RIN3 promotes β-secretase processing of APP. However, BIN1-expression was not shown to alter APP-processing in PC12 cells^16^. Although RIN3 has been identified as a genetic risk factor for AD, how increased expression of RIN3 in AD potentially contributes to AD pathogenesis remains unclear.

Here, we have employed in vitro studies to demonstrate that RIN3 translocates neuronal BIN1V1, but not non-neuronal BIN1V9, to RAB5-positive early endosomes. RIN3-mediated recruitment of BIN1V1 to RAB5-positive early endosomes was independent of BIN1-RIN3 binding or RAB5-activation. We next showed that BIN1V1 significantly reduces β-secretase cleavage of APP, in a RIN3-dependent manner. In contrast, non-neuronal BIN1V9 did not alter β-secretase mediated processing of APP. We next performed internalization and Aβ ELISA assays^26^, and found that BIN1V1 delays the endocytosis of APP (but not of BACE1) into early endosomes, separating these two proteins into different cellular compartments thereby attenuating Aβ generation, in a RIN3-dependent manner. Overall, our results show that RIN3 sequesters BIN1V1 in RAB5-positive early endosomes, likely via the CLAP-domain, resulting in attenuated β-secretase processing of APP and Aβ generation owing to delayed endocytosis of APP. Overall, our findings strongly indicate that the neuronal isoform BIN1V1, but not the non-neuronal isoform BIN1V9, regulates APP-processing and Aβ generation in a RIN3-dependent manner.

## Materials and methods

### Cell lines, chemicals, and antibodies

HEK293 and Neuro 2A (N2A) cells were grown in DMEM (Gibco/Life Technology) media containing 10% fetal bovine serum (FBS) and 10U/ml penicillin, 100 μg/ml streptomycin. N2A cells constitutively expressing APP_751_ (N2A_APP_) were used to measure β-secretase processing of APP and Aβ generation. N2A_APP_ cells were prepared by transfecting expression plasmid containing cDNA encoding APP_751_ (pcDNA3-APP_751_). Stable cells were selected in presence of G418 as described before^27^. Stable cells were maintained in DMEM media (Lonza) containing 10% FBS, 100 U/ml penicillin, 100 μg/ml streptomycin, 2 mM L-glutamate supplemented with 200 μg/ml G418.

The following APP-antibodies were used to detect full-length and APP-metabolites: 22C11 (anti-APP N-terminus, Millipore), anti-sAPPβ (IBL International). Anti-BIN1 (Abcam) and anti-RIN3 antibodies were used to detect BIN1_GFP_ and RIN3_flag_ expressions. Anti-flag (Abcam) was used to detect RIN3_flag_. Anti-GAPDH (Life Technologies) was used to determine GAPDH expression as loading control. Anti-transferrin receptor (anti-Tfr, Abcam) was used to confirm cell surface biotinylation. Alexa Fluor (488, 568 or 350)- or HRP-conjugated secondary antibodies were purchased from Life Technologies. BIN1V1_GFP_ and BIN1V9_GFP_ expression were visualized by direct fluorescence microscopy. Expression plasmids for BIN1V1_GFP_ and BIN1V9_GFP_ were generous gifts from Dr. Patrik Verstreken, Janssen Pharmaceutical, Belgium. The expression plasmid for RIN3_flag_ was obtained from Dr. Toshiaki Katada (University of Tokyo). Expression plasmids encoding mCherry-epitope tagged RAB5 or RAB5QL were purchased from Adgene.

### Cell surface biotinylation and internalization assay

The cell surface trafficking assay was performed following published method^28^. The endocytosis assay was performed according to published protocol^29^. Briefly, cells were starved for 16 h prior to labeling with 0.5 mg/ml cell-impermeable sulfo-NHS-SSBiotin (Pierce) at 4 °C for 1 h. Cells were then moved to 37 °C for appropriate time (0, 10, or 30 min) to allow endocytosis before returning to 4 °C. Remaining biotin at the cell surface were cleaved off by 100 mM 2-sodium-2-mercaptoethanesulfonate (Sigma). Proteins were extracted in extraction buffer containing 10 mM Tris (pH 7.4), 2 mM EDTA, 150 mM NaCl, 1% Triton X-100, 0.5% sodium deoxycholate, 0.2% SDS, and protease inhibitors. Approximately 750 μg of cell extract was subjected to pull-down with Neutravidin beads to purify biotinylated cell surface proteins followed by incubation with SDS sample buffer containing 0.1% β-mercaptoethalol (βME). Pulled-down samples were probed with appropriate antibodies to detect the levels of internalized APP and BACE1 by measuring the band intensities of the respective proteins after 0, 10 and 30 min of endocytosis.

To assess APP endocytosis by confocal microscopy, cells were placed at 4 °C in pre-chilled media containing 22C11 (anti-N terminus APP) for 1 h to capture cell surface APP. Next, cells were moved to 37 °C for endocytosis, which was stopped by again moving the cells to 4 °C after 0, 10 and 30 min. Cells from each time points were fixed, permeabilized (0.1% Triton in PBS, 5 min at RT) and prepared for confocal microscopy by labeling with secondary antibody containing Alexa Fluor 568.

### Immunostaining

Immunostaining is performed by following methods described before^27,30^. Briefly, transfected cells were fixed with 4% paraformaldehyde (PFA) for 20 min at room temperature. 3% PFA was made in PBS containing calcium and magnesium. After washing three-times with PBS cells were blocked using Blocking Solution (1% BSA, 0.1% gelatin, 0.1% Triton X-100, 0.05% Tween-20 in PBS containing calcium and magnesium). Cells were then labeled with appropriate primary antibody solutions (1:250) for 1 h prior to washing and incubating with secondary antibody (1:250 dilution) conjugated with appropriate Alexa fluorophores for 45 min. followed by confocal microscopy as done before^30^. Proteins conjugated with fluorophores, *eg.* BIN1V1/V9_GFP_ or RAB5_mCherry_, were imaged directly because the fluorophores have provided sufficiently strong signals.

### Confocal microscopy

Subcellular distribution of RIN3_flag_, or APP were detected by indirect immunostaining of the cells following methods described before^31^. Expression of BIN1V1_GFP,_ BIN1V9_GFP_, or RAB5_mCherry_ were visualized by direct confocal microscopy. Briefly, cells were fixed in 4% paraformaldehyde (PFA) prior to labeling with antibody against APP (22C11) followed by appropriate fluorescence conjugated secondary antibodies. Fluorescence microscopy was performed under Nikon confocal microscope using 40X objective. Images were processed by ImageJ software to determine co-localization.

### Co-localization analysis

To quantitate co-localization of BIN1V1 or BIN1V9 with RIN3_flag_, we imaged cells co-expressing BIN1V1_GFP_ + RIN3 or BIN1V9_GFP_ + RIN3. BIN1V1/V9 were imaged directly, while RIN3 expression was detected by labeling cells with anti-flag antibody followed by secondary antibody conjugated with Alexa-488 red fluorescent dye. Colocalized area (in yellow) and total area (in green) of cells expressing BIN1V1 or BIN1V9 were measured. A ratio between the co-localized area and total area was converted to percent co-localization.

To quantitate co-localization of BIN1V1 or BIN1V9 with RAB5-positive endocytic vesicles in cells overexpressing RIN3, we performed colocalization analysis as described before^32,33^, with modifications. Briefly, cells expressing BIN1V1_GFP_/BIN1V9_GFP_ (green fluorescent) and RAB5_mCherry_ (red fluorescent) were imaged directly, while RIN3_flag_ expression was determined by labeling cells with anti-flag antibody followed by secondary antibody conjugated with Alexa Cy5 (blue fluorescent) fluorescent dye. Confocal images of the cells were imported into the Fiji version of the free software ImageJ. Fluorescent images of single cells were opened and were split. The green and the red channels were subjected to pre-installed Coloc2 plugins analyses to calculate the colocalization parameter, Pearson coefficient (r). The values were converted to percent to show the percent of BIN1V1 or BIN1V9 co-localization with RAB5.

### FACS enrichment of the transfected cells

N2A_APP_ cells expressing either BIN1V1_GFP_ or BIN1V1_GFP_ + RIN3_flag_ were subjected to FACS sorting to enrich cells expressing the indicated proteins. Briefly, 24 h post-transfected cells were resuspended in PBS supplemented with 2% serum replacement solution (Life Technologies) and 2% B27, and then passed through a cell strainer filter (70 mm Nylon, BD Biosciences). The cell concentrations were adjusted to ~ 200,000 cells per ml. Single cells were identified by size (forward scatter laser light) and granularity (90 degrees side scatter laser light). The cells with GFP fluorescence were deflected, and collected with a BDFACSAria Fusion Cell Sorter (BD Biosciences, San Jose, CA). (MGH core facility, Charlestown, MA), as described before^31^. GFP-positive cells were collected in 5 ml or 15 ml tube with 2/3 ml of DMEM media with 2% FBS or BSA. The sorted/enriched cells were maintained for 48–72 h. The media was exchanged with fresh media 24 h prior to measuring sAPPβ and Aβ release from the FACS sorted cells.

### ELISA and western blot

Aβ (Aβ_40_ and Aβ_42_) species were measured from conditioned media of FACS sorted cells using commercially available ELISA kits from WAKO, as described before^27,31^. Protein concentrations were determined by Bio Rad BCA assay. For our assays, we transfected 1 × 10^6^ cells with appropriate expression plasmids using Effectene (Qiagen) transfection reagent following manufacturer’s protocol. After 18 h transfection the conditioned media (CM) were collected to determine the levels of soluble APP fragments (sAPPβ, total sAPP or sAPP_tot_, and sAPPα) or Aβ as routinely done in our laboratory^27,30^. Total Cell lysates (TCL) were prepared in extraction buffer (10 mM Tris–HCl, pH 7.6, 1% Triton-100, 150 mM NaCl, 2 mM EDTA, 2.5% NP40, containing protease inhibitor cocktail) as described before^27^. For Western blot analysis samples were prepared in sample buffers containing β-mercaptoethanol (βME) for denaturation. Equal amounts of proteins (~ 30 μg) were loaded on NuPAGE 4–12% Bis–Tris gel (Invitrogen) for electrophoresis and Western blot analysis as described before^27^. Unless otherwise specified, we used 1:1000 and 1:6000 dilutions for the primary and the HRP-conjugated secondary antibodies, respectively, were used for Western blot assays. The blots were visualized by a Li-Cor Odyssey imaging system. Band intensities were measured by ImageStudio software. Western blots were stripped using stripping buffer (6.25 mM Tris–HCl, pH 6.8, 0.2% SDS, 0.8% β-mercaptoethanol) for 30 min at 50 °C followed by thorough washing with PBS (5X for 15 min each) prior to re-probing the blot with different antibodies. Uncropped images of all important Western blots are presented in supplementary figures. Equal amounts of some samples, mostly total cell lysates (TCL) or total inputs, were occasionally subjected to electrophoresis on separate gels to avoid background in the Western blots after going through two or more stripping processes. The uncropped gets of TCLs and inputs are available upon request. Levels of GAPDH was used as loading control. However, in most cases total APP levels indicated equal loading.

### Statistical analysis

All statistical analyses were performed using a two-way ANOVA or two-tailed Student’s t-test. Data in graphs are expressed as mean values SEM. Calculations were performed either by Microsoft Excel or by GraphPad Prism. *p* < 0.01 was considered significant.

## Results

### RIN3 recruits BIN1V1, but not BIN1V9, to RAB5-positive endocytic vesicles

**Figure 1:**
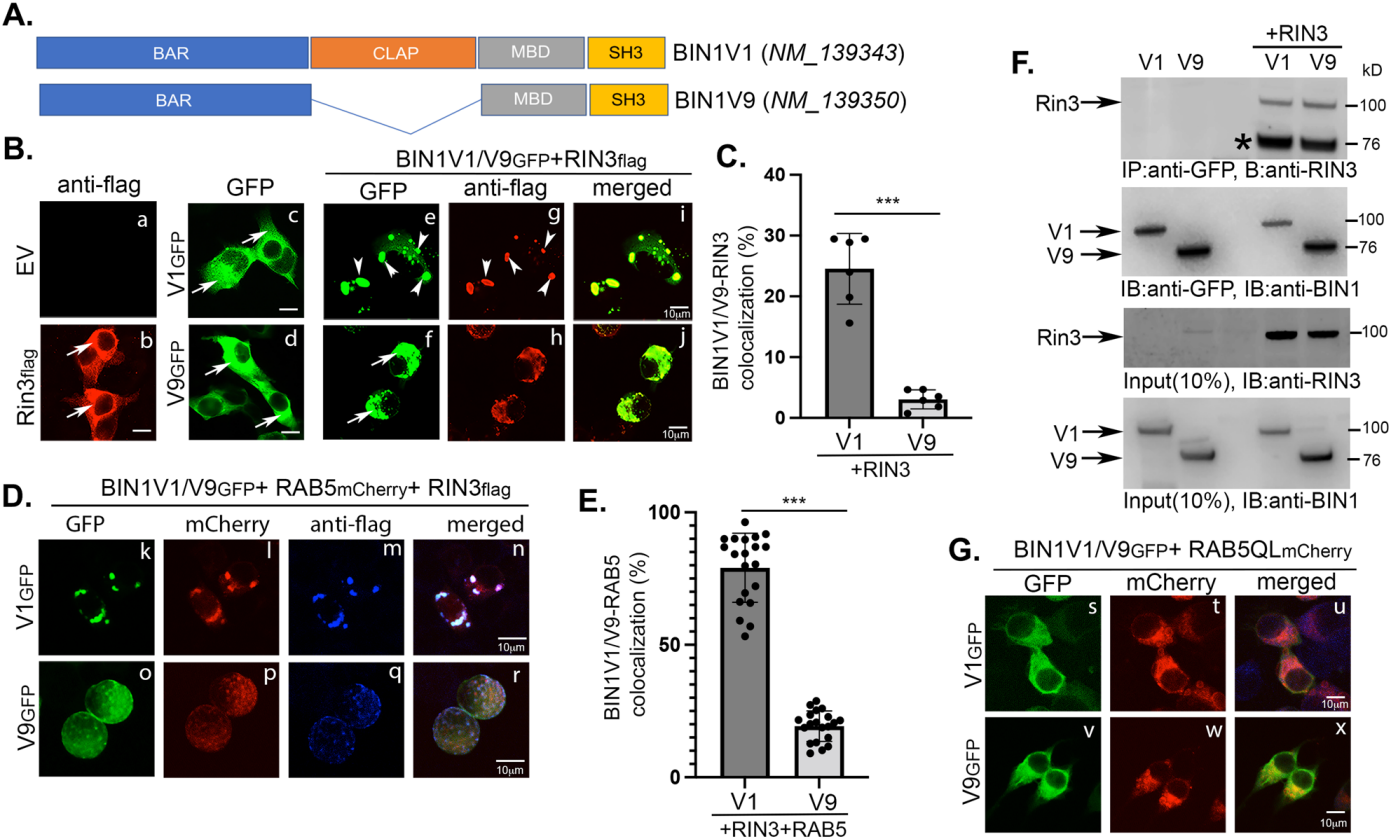
The neuronal isoform BIN1V1, but not non-neuronal BIN1V9 translocates to RAB5-positive early endosomes in a RIN3-dependent manner. (**A**) Schematics of neuronal BIN1V1 and non-neuronal BIN1V9 proteins. BAR (Bin-Amphiphysin-Rvs), CLAP (clathrin and AP-2-binding), MBD (Myc-binding domain) and SH3 (Src homology domain 3) domains are indicated. BIN1V1 contains the CLAP domain, while BIN1V9 does not. (**B**) Confocal microscopy of neuro 2A cells expressing control empty vector (EV, a), flag-epitope tagged RIN3 (RIN3_flag_, a), BIN1V1_GFP_ (V1_GFP_, c and e) or BIN1V9_GFP_ (V9_GFP_, d and f) without (c and d) or with RIN3_flag_ (e-j). Cells were fixed and immunostained with anti-flag antibody to detect RIN3_flag_ (anti-flag) distribution. GFP-signals (GFP) detected subcellular distribution of GFP or BIN1V1/V9_GFP_. Confocal microscopy shows predominant cytoplasmic distribution (arrows) of RIN3_flag_, BIN1V1_GFP_ and BIN1V9_GFP_, when expressing alone (b, c, and d, respectively). BIN1V1_GFP_ and RIN3_flag_ translocated to endocytic vesicles when co-expressed (arrowheads, e, and g). BIN1V9_GFP_ and RIN3_flag_ remained cytoplasmic even when expressed together (arrows, f, and h). Merged images (merged, i and j). (**C**) Analysis of BIN1V1 or BIN1V9 co-localization with RIN3, V1 + RIN3 or V9 + RIN3, respectively. 6 frames containing 5–10 cells were measured. ***p < 0.001. (**D**) Confocal microscopy of cells expressing BIN1V1_GFP_ or BIN1V9_GFP_ with RIN3_flag_ (anti-flag) and RAB5_mCherry_ (mCherry). BIN1V1_GFP_ and RIN3_flag_ co-localized with RAB5_mCherry_ in the RAB5-positive early endosomes (V1_GFP_, k-n). BIN1V9_GFP_ remained cytoplasmic in presence of RIN3 (V9_GFP,_ o-r). (**E**) Analysis of BIN1V1 or BIN1V9 co-localization with RAB5 in cells overexpressing RIN3. *n* = 25, ***p < 0.001. (**F**) Representative immunoblot (IB) of co-immunoprecipitation assay where cells expressing BIN1V1_GFP_ (V1), BIN1V9_GFP_ (V9), BIN1V1_GFP_ + RIN3_flag_ (V1 + RIN3) or BIN1V9_GFP_ + RIN3_flag_ (V9 + RIN3) were subjected to immunoprecipitation (IP) using anti-GFP antibody. Immunoprecipitated (IPed) samples were probed with anti-BIN1 antibody (IP:anti-GFP, IB:anti-BIN1) to demonstrate immunoprecipitation of BIN1V1_GFP_ (V1) and BIN1V9_GFP_ (V9). IPed samples were probed with anti-RIN3 antibody (IP: anti-GFP, IB: anti-RIN3) to determine RIN3 binding with BIN1V1 or BIN1V9. Immunoprecipitation with anti-GFP antibody pulled down RIN3 (~ 100kD) from cells co-expressing BIN1V1 + RIN3_flag_ or BIN1V9_GFP_ + RIN3_flag_. Anti-RIN3 antibody consistently detected a non-specific band at ~ 76kD. (**G**) Confocal microscopy of cells expressing BIN1V1_GFP_ (V1_GFP_, s) or BIN1V9_GFP_ (V9_GFP_, v) with constitutively active RAB5QL_mCherry_ (mCherry, t and w).

Among the isoforms of BIN1, neuronal BIN1V1 containing the CLAP domain and non-neuronal BIN1V9 lacking the CLAP domain are the major isoforms present in the brain (Fig. 1A). Several reports have shown that BIN1V1 levels are increased in AD, while the levels of BIN1V9 are decreased. In vitro studies have shown that the BIN1-binding partner, RIN3, recruits the BIN1 paralog, Amphiphysin II, to RAB5-positive early endosomes via direct interaction with the N-terminal SH3 domain of BIN1 (Fig. 1A), initiating endocytosis^18^. To test whether RIN3 recruits both major brain isoforms of BIN1 (BIN1V1 and BIN1V9) to RAB5-endosomes, we compared RIN3-mediated redistribution of BIN1V1 and BIN1V9 in Neuro 2A (N2A) cells. Confocal microscopy of cells expressing flag-epitope tagged RIN3 (RIN3_flag_), or GFP-epitope tagged BIN1V1 (BIN1V1_GFP_) or BIN1V9 (BIN1V9_GFP_) showed predominant cytoplasmic distribution of RIN3_flag_, BIN1V1_GFP_ and BIN1V9_GFP_ (Fig. 1A, b, c, and d, respectively). Cells co-expressing RIN3_flag_ and BIN1V1_GFP_ (RIN3_flag_ + BIN1V1_GFP_) exhibited strong translocation of both BIN1V1_GFP_ and RIN3_flag_ to the endocytic vesicles (Fig. 1B, e and g, respectively), as expected. However, cells co-expressing RIN3_flag_ and BIN1V9_GFP_ (RIN3_flag_ + BIN1V9_GFP_) showed predominantly cytoplasmic distribution of BIN1V9_GFP_ (Fig. 1B, f and h). Quantitation of BIN1V1/BIN1V9 colocalization with RIN3 revealed that while 24.5 ± 5.8% BIN1V1 colocalized with RIN3, primarily in the endocytic vesicles, only 3.1 ± 1.5% BIN1V9 exhibited co-localization with RIN3 (Fig. 1C). Thus, BIN1V9 showed nearly 88% reduced co-localization with RIN3 compared to BIN1V1. By co-transfecting a plasmid encoding mCherry epitope tagged RAB5 (RAB5_mCherry_), we confirmed that RIN3 recruited BIN1V1 to the RAB5-positive early endosomes (Fig. 1D, k-n). However, BIN1V9_GFP_ remained largely cytoplasmic in cells expressing RIN3_flag_ and RAB5_mCherry_ (Fig. 1D, o-r). Pearson’s coefficient (r) analysis (Supplementary Figure S1) followed by quantitation revealed that while 79.06 ± 13.03% BIN1V1 colocalized with RAB5-positive endocytic vesicles, only 19.31 ± 7.75% BIN1V9 exhibited co-localization with RAB5 (Fig. 1E). Thus, our confocal image analysis showed that RIN3 recruits neuronal BIN1V1 to RAB5 endosomes by nearly fourfold more efficiently than BIN1V9. Similar results were obtained in HEK 293 cells overexpressing BIN1V1, BIN1V9 without or with Rin3 (data not shown).

Next, we asked whether RIN3-dependent recruitment of BINV1, but not BINV9, to RAB5 endosomes was due to the differences in binding affinity of the two isoforms for RIN3. To this end we performed a co-immunoprecipitation assay in cells expressing BIN1V1_GFP_ or BIN1V9_GFP_ without or with RIN3_flag_ (Fig. 1F and Supplementary Figure S2). Both BIN1V1_GFP_ and BIN1V9_GFP_ pulled down RIN3_flag_, suggesting equal affinity of both isoforms of BIN1 (BIN1V1 and BIN1V9) towards RIN3 (Fig. 1F, IP: anti-BIN1, IB: anti-RIN3). This is not surprising because RIN3 binds to BIN1 via the N-terminal SH3 domain that is present in both BIN1V1 and BIN1V9. Thus, RIN3-mediated recruitment of BIN1V1 to the RAB5-positive early endosomes was independent of RIN3-binding. Intriguingly, BIN1V9 does not contain the CLAP domain, while BIN1V1 does. This suggests that the CLAP-domain may be required for RIN3-mediated recruitment of BIN1 to RAB5-positive early endosomes.

RIN3 is a guanine nucleotide exchange factor (GEF) for RAB5, which activates RAB5 by exchanging GDP for GTP^19^. To test whether RAB5-activation via RIN3 translocates BIN1V1 to RAB5-positive endosomes, we assessed the subcellular distribution of BIN1V1_GFP_ in presence of an mCherry-labeled RAB5 mutant containing a Gln^79^ (Q) to Leu(L) substitution (RAB5QL_mCherry_). Previous reports showed that the Q^79^L substitution reduces GTPase activity of RAB5 to maintain RAB5QL in a constitutively active state^34^. Confocal images showed that expression of RAB5QL_mCherry_ had little or no effect on the cytoplasmic distribution of BIN1V1_GFP_ (Fig. 1G, s-u), suggesting that RIN3-mediated endosomal-recruitment of BIN1V1 was independent of RAB5-activation. RAB5QL also did not recruit BIN1V9_GFP_ to the endocytic vesicles (Fig. 1G, v-x). Overall, our results show that RIN3-mediated recruitment of BIN1V1 to the early endosomes is dependent on the CLAP-domain, but independent of the RIN3-BIN1 binding or of RAB5-activation.

### Neuronal BIN1V1, but not non-neuronal BIN1V9, reduces β-secretase cleavage of APP in a RIN3-dependent pathway

The role of BIN1 in APP-processing or Aβ generation is not clear. Previous reports showed that RNAi-mediated silencing of BIN1 expression increases Aβ production without changing sAPPβ generation^35^, while overexpression of BIN1 showed no effect on APP-processing *in vitro*^16^. Another recent in vitro study showed that overexpression of RIN3 increased APP processing^35^. Here, we explored whether the varying effect of RIN3 on the subcellular distribution of the two BIN1 brain isoforms results in differential effects on β-secretase cleavage of APP, the rate-limiting step for Aβ generation.

**Figure 2:**
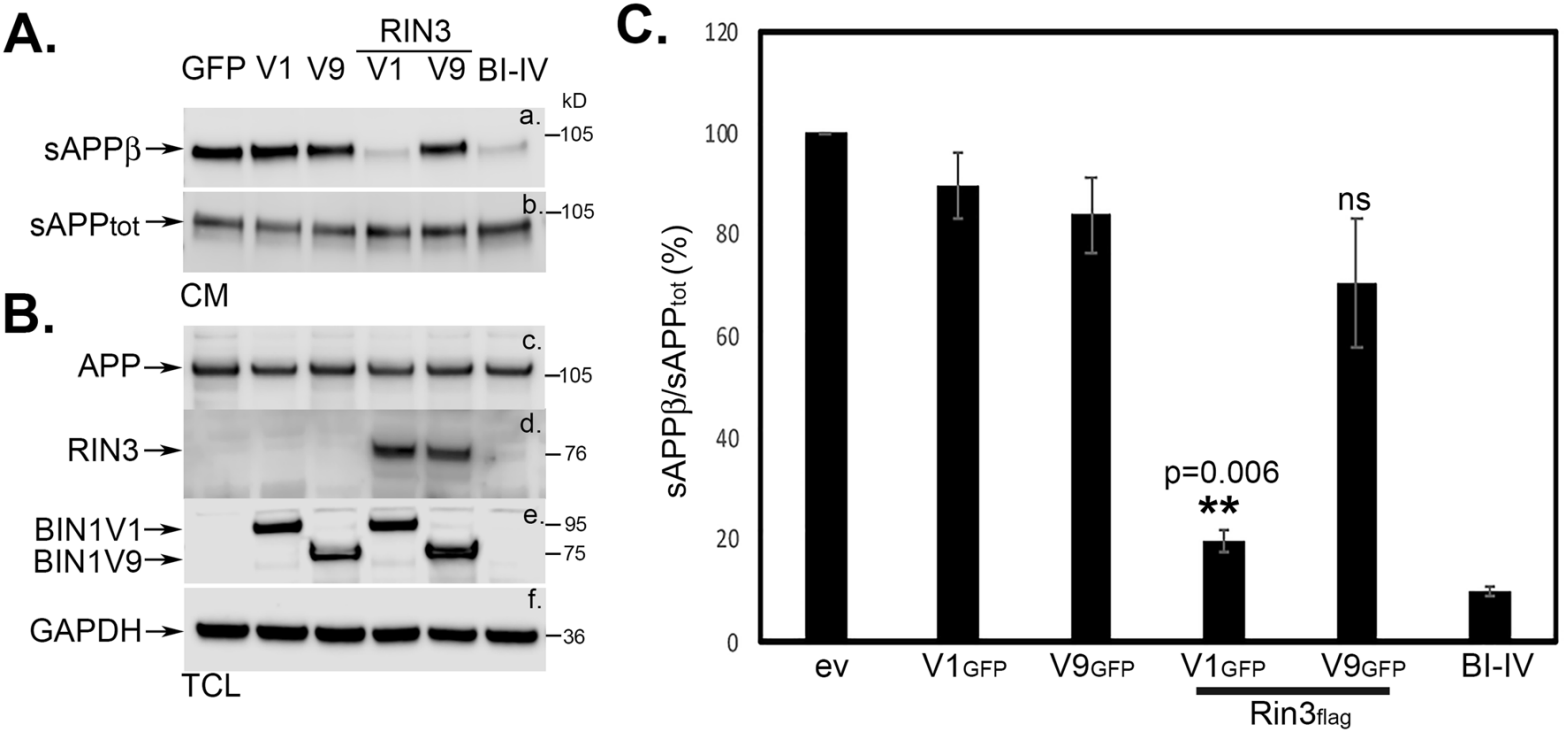
The neuronal isoform BIN1V1, but not the non-neuronal BIN1V9, reduces β-secretase-mediated processing of APP in a RIN3-dependent manner. **(A)** Representative immunoblot image of the conditioned media (CM) or total cell extracts (TCL) of FACS enriched N2A_APP_ cells overexpressing GFP (GFP), BIN1V1_GFP_ (V1), BIN1V9_GFP_ (V9), BIN1V1_GFP_ + RIN3_flag_ (V1 + RIN3) or BIN1V9_GFP_ + RIN3_flag_ (V9 + RIN3). sCM were probed with anti-sAPPβ (a.) or 22C11 (b.) to detect sAPPβ or sAPP_tot_, respectively. CM from cells pre-treated with 5 μM BACE1 inhibitor IV (BI-IV) for 16 h was also probed to confirm detection of sAPPβ. Total cell lysates (TCL) were probed with anti-APP 22C11 (c.), anti-RIN3 (d.), anti-BIN1 (e), and anti-GAPDH (f.) to detect APP, RIN3_flag_, BIN1V1_GFP_/BIN1V9_GFP_, and GAPDH, respectively. (**B)** Quantitation of sAPPβ release (anti-sAPPβ) compared to sAPP_tot_ (22C11) from the cells expressing indicated proteins. Error bars represented ± SEM from three independent experiments. **p < 0.01.

For this purpose, we compared the effects of BIN1V1 versus BIN1V9 on β-secretase processing of APP, in the presence or absence of RIN3. To measure β-secretase cleavage of APP, we assessed soluble APPβ (sAPPβ) levels in the conditioned media (CM) of APP_751_-expressing Neuro 2A cells (N2A_APP_). First, we expressed GFP, BIN1V1_GFP_ (V1_GFP_), BIN1V9_GFP_ (V9_GFP_), BIN1V1_GFP_ + RIN3_flag_ (V1_GFP_ + RIN3_flag_) or BIN1V9_GFP_ + RIN3_flag_ (V9_GFP_ + RIN3_flag_) in N2A_APP_ cells. Cells were then sorted by a fluorescence-activated cells sorter (FACS), as previously described^31^, to obtain homogenous cultures of the cells expressing the GFP-tagged proteins. FACS sorted cells were grown to confluency before collecting conditioned media (CM) to measure total sAPP (sAPP_tot_), sAPPβ, and sAPPα levels (Fig. 2A and Supplementary Figure S3). Cells co-expressing BIN1V1_GFP_ + RIN3_flag_ exhibited a significant reduction in sAPPβ release compared to cells overexpressing GFP or BIN1V1_GFP_ alone (Fig. 2A, CM, IB: anti-sAPPβ). Overexpression of BIN1V9_GFP_ or BIN1V9_GFP_ + RIN3_flag_ resulted in little or no change of sAPPβ release compared to control cells (GFP) (Fig. 2A, CM, IB: anti-sAPPβ). The CM were also probed with the antibodies that recognized total sAPP (sAPP_tot_) (22C11). We observed that overexpression of BIN1V1 or BIN1V9, with or without RIN3, had little or no effect on sAPP_tot_ level in the CM (Fig. 2A, CM). Quantitation revealed that cells co-expressing BIN1V1 and RIN3 (BIN1V1_GFP_ + RIN3_flag_) decreased sAPPβ release by ~ 66% (0.34 ± 0.093-fold (*p* = 0.0543, *n* = 3)) versus GFP-expressing control (GFP) cells (Fig. 2B). The changes in the levels of sAPPβ from cells overexpressing BIN1V1_GFP_ (V1_GFP_), BIN1V9_GFP_ (V9_GFP_), or BIN1V9_GFP_ + RIN3_flag_ (V9_GFP_ + RIN3_flag_) were not significant as compared to sAPPβ levels released from control (GFP) cells (Fig. 2B). Our results demonstrate that neuronal BIN1V1, but not BIN1V9, reduces β-secretase-mediated processing of APP in a RIN3-dependent manner. Taken together, these results indicate that BIN1-overexpression reduces β-secretase processing of APP exclusively in the neurons, in a RIN3-dependent manner.

### BIN1V1 delays the internalization of APP in a RIN3-dependent manner

**Figure 3:**
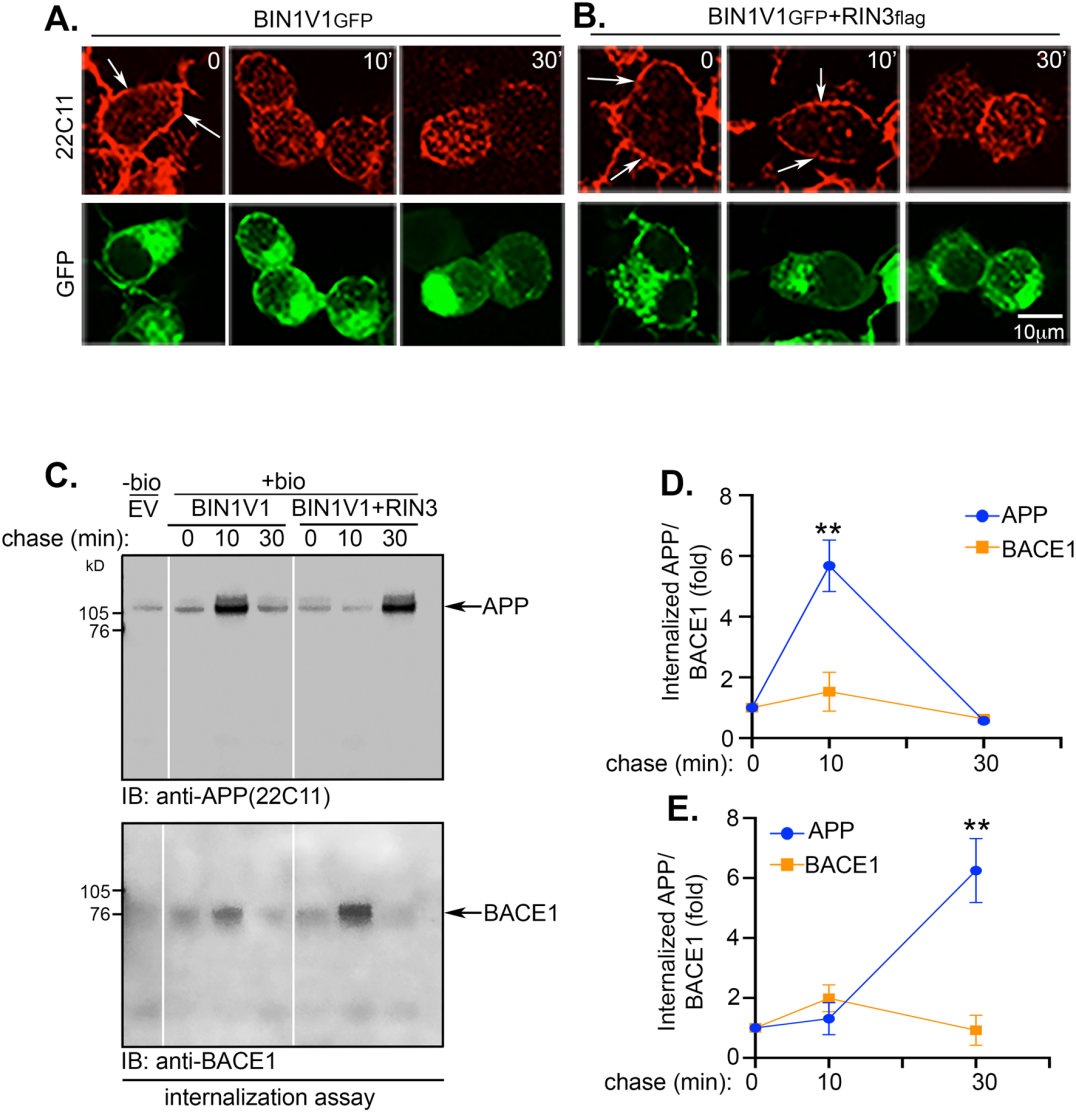
Co-expression of RIN3 with the neuronal isoform BIN1V1 delays cell surface endocytosis of APP. (**A)** and (**B)** Confocal images of N2A_APP_ cells overexpressing BIN1V1_GFP_ or BIN1V1_GFP_ + RIN3_flag_ labeled with 22C11 at 4 °C for 1 h to allow labeling cell surface APP followed by endocytosis at 37 °C for 0, 10 and 30 min as before. Loss of cell surface APP (arrows) suggested endocytosis. Representative images of approximately 20 cells per experiment performed in duplicates. (**C)** Representative Western blot images of internalized biotinylated APP (IB:22C11) or BACE1 (anti-BACE1) after 0-, 10- and 30-min chase of N2A_APP_ cells overexpressing BIN1V1_GFP_ or BIN1V1_GFP_ + RIN3_flag_. (**D)** and (**E)** Graphs are quantitation of internalized biotinylated APP (blue circles) or BACE1 (orange squares) after 0-, 10- and 30-min chase of cells overexpressing BIN1V1_GFP_ (**D**) or BIN1V1_GFP_ + RIN3_flag_ (**E**). Error bars represented ± SEM from three independent experiments. *n* = 3, Two-way ANOVA statistical analysis provides **p < 0.005.

Previous studies have shown that APP is internalized from the plasma membrane in RAB5-positive endosomes and then undergoes β-secretase cleavage^36,37^. Here, we examined whether RIN3 and BIN1V1 alter APP-internalization and subsequent cleavage of APP by β-secretase. To visualize APP internalization, we performed confocal microscopy by first live feeding N2A_APP_ cells with anti-APP N-term antibody (22C11) for 1 h at 4 °C to label cell surface APP followed by 10 min and 30 min chase at 37 °C. Cells were then subjected to indirect immunofluorescence to detect the loss of cell surface APP as a measure of APP internalization. Both BIN1V1_GFP_ or BIN1V1_GFP_ + RIN3_flag_ expressing cells showed strong cell surface staining of APP prior to the chase (Fig. 3A and B, arrows), as expected. Cells overexpressing BIN1V1_GFP_ exhibited a significant loss of cell surface APP within 10 min chase (Fig. 3A, 10’), suggesting that cell-surface APP internalized within 10 min. As expected, 30 min of chase resulted in a complete loss of cell surface APP (Fig. 3A, 30’). In contrast, cells overexpressing both BIN1V1_GFP_ and RIN3_flag_ showed strong cell surface labeling of APP even after 10 min of chase (Fig. 3B, 10’, arrows), suggesting delayed internalization. Loss of cell surface APP was visualized after 30 min of chase (Fig. 3B, 30’). Cells co-expressing BIN1V1 and RIN3 were identified by the signature punctate distribution pattern of BIN1V1_GFP_ in these cells as compared to the predominant cytoplasmic distribution of BIN1V1_GFP_ observed in cells overexpressing BIN1V1_GFP_ alone (Fig. 3B, GFP). Thus, our confocal microscopy results suggest delayed endocytosis of APP in cells co-expressing RIN3 and BIN1V1 as compared to cells expressing BIN1V1, alone.

The colocalization of APP and BACE1 in early endosomes is essential for β-secretase cleavage of APP and Aβ generation^29,38^. To test whether the delayed internalization of APP in cells co-expressing BIN1V1 and RIN3 resulted in impaired β-secretase cleavage of APP by preventing colocalization of APP with BACE1 in early endosomes, we assessed the rates of APP and BACE1 internalization. We labeled cell surface proteins with a cell impermeable biotinylating agent followed by chasing the internalization of the biotinylated surface proteins for 10 min and 30 min at 37 °C (Fig. 3C, chase(min): 0, 10 and 30). The internalized biotinylated proteins were then pulled down by neutravidin beads after stripping excess biotin from the cell surface. The biotinylated proteins that were pulled down were probed for APP and endogenous BACE1 to assess their internalization rates. We observed a strong increase in the levels of both APP and endogenous BACE1 within 10 min of chase in un-transfected (not shown) cells, as well as in cells expressing BIN1V1_GFP_ alone (Fig. 3C, 10 min), suggesting that APP and BACE1 were internalized within 10 min in absence or presence of BIN1V1. After 30 min chase, we observed robust reduction in the levels of internalized APP and endogenous BACE1 (Fig. 3C, 30 min), suggesting degradation in late endosomes or lysosomes, as expected^25^. Interestingly, the kinetics of APP internalization were distinctly different from those of BACE1 in cells co-expressing BIN1V1_GFP_ + RIN3_flag_ (Fig. 3C, and Supplementary Figure S4). APP was internalized only after 30 min chase, while endogenous BACE1 was internalized within 10 min, in cells co-expressing BIN1V1_GFP_ and RIN3_flag_. These data suggest delayed internalization of APP, but not of BACE1, in cells over expressing BIN1V1 and RIN3 compared to cells expressing BIN1V1, alone (Fig. 3C, IB:22C11). Quantitation revealed that a 10 min chase resulted in 7.68 ± 1.04-fold increase in the levels of internalized APP in cells expressing BIN1V1 alone, while 30 min of chase were required to achieve a 6.68 ± 1.05-fold increase of internalized APP, in cells co-expressing both BIN1V1 and RIN3 (Fig. 3D, E). In contrast, the levels of internalized BACE1 reached ~ twofold within 10 min chase in both BIN1V1 and BIN1 + RIN3 expressing cells (Fig. 3D, E). Chasing for 10 and 30 min did not result in any significant change of total levels of APP or BACE1 (Supplementary Figure S4, *Total Input*). These results not only confirmed our confocal microscopic data that APP-internalization was delayed upon overexpression of RIN3 with BIN1V1, but also showed that BACE1-internalization remains unaltered under these conditions. Together, our results indicate that BIN1V1 delays endocytosis of APP, but not of BACE1, in a RIN3-dependent manner, leading to spatial and temporal separation of APP from BACE1 and reduced cleavage of APP by β-secretase in early endosomes. Additionally, internalization rate of endogenous transferrin receptor (Tfr) also remained unaltered in cells expressing BIN1V1_GFP_ or BIN1V1_GFP_ + RIN3_flag_ (Supplementary Figure S4, IB:anti-Tfr). Although, this result provides an additional proof for RIN3-dependent delay of APP internalization in BIN1V1-expressing cells compared to that of Tfr, a more detailed study is required in future to clearly assess the differences between the RIN3-dependent internalizational of Tfr in comparison to APP.

### Co-expression of RIN3 with BIN1V1 reduces the generation Aβ

**Figure 4:**
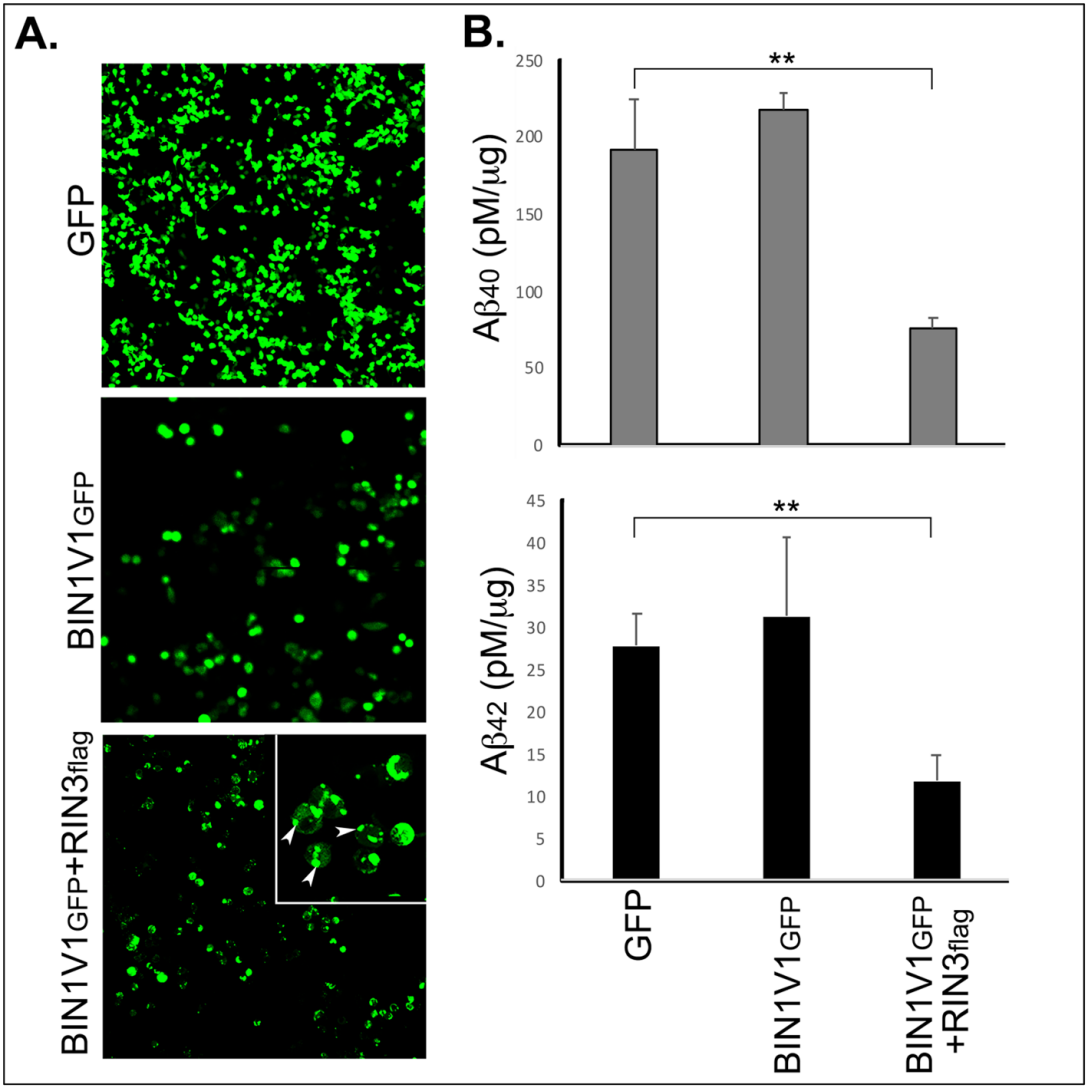
Co-expression of RIN3 with the neuronal isoform BIN1V1 in N2A_APP_ cells reduces Aβ generation. (**A)** Representative confocal image of N2A_APP_ cells expressing GFP, BIN1V1_GFP_ or BIN1V1_GFP_ + RIN3_flag_. GFP and BIN1_GFP_ exhibited predominant cytoplasmic distribution. BIN1V1_GFP_ distributed in discrete puncta (*Inset*, arrowheads) in cells when co-expressed with RIN3_flag_ (BIN1V1_GFP_ + RIN3_flag_). (**B)** Condition media of FACS sorted cells overexpressing BIN1V1_GFP_ or BIN1V9_GFP_ + RIN3_flag_ were subjected to Aβ ELISA assay to measure Aβ_40_ and Aβ_42_ levels. Error bars represented ± SEM from three independent experiments. ** represents *p* < 0.01.

Next, we tested whether BIN1V1 reduces Aβ generation in a RIN3-dependent manners. For this purpose, we compared Aβ (Aβ_40_ and Aβ_42_) release from N2A_APP_ cells overexpressing BIN1V1_GFP_ or BIN1V1_GFP_ + RIN3_flag_. To maximize the number of cells overexpressing BIN1V1_GFP_ or BIN1V1_GFP_ + RIN3_flag_, we subjected the overexpressing cells to FACS sorting (Fig. 4A)^31^. The FACS sorted cells (Supplementary Figure S5) were grown to confluency (Fig. 4A). The signature punctate distribution of BIN1V1_GFP_ in > 90% cells co-expressing BIN1V1_GFP_ and RIN3_flag_ confirmed that RIN3_flag_ was expressed in the FACS sorted cells (Fig. 4A, arrowheads). Aβ ELISA of the conditioned media (CM) of cells overexpressing BIN1V1_GFP_ revealed no effect on Aβ (Aβ_40_ and Aβ_42_) generation as compared to the GFP-expressing control cells (Fig. 4B). In contrast, cells co-expressing RIN3 with BIN1V1 (BIN1V1_GFP_ + RIN3_flag_) resulted in a dramatic reduction in both Aβ_40_ and Aβ_42_ levels (Fig. 4B). Quantitation revealed that BIN1V1_GFP_ alone led to 217.36 ± 12.13 pM/μg Aβ_40_ and 31.27 + 9.32 pM/μg Aβ_42_, while cells co-expressing BIN1V1_GFP_ and RIN3_flag_ generated 76.72 ± 6.31 pM/μg Aβ_40_ and 11.72 ± 3.11 pM/μg of Aβ_42_. Overall, these data show that overexpression of BIN1V1 attenuates Aβ generation in a RIN3-dependent manner.

## Discussion

Prior studies have shown that the AD genetic risk factor, BIN1, is involved in regulating RAB5-mediated endocytosis to modulate both Aβ and tau pathology in AD, by either “freeing” or “sequestering” the BIN1 binding partner and RAB5 effector, RIN3^17,39^. However, to date, no direct role of RIN3 on BIN1-mediated effects on AD pathogenesis has been demonstrated. Here, we show that neuronal-specific isoform of BIN1, BINV1, but not the non-neuronal isoform, BIN1V9, regulates APP trafficking, processing of APP by β-secretase (BACE1), and Aβ generation in a RIN3-dependent manner. Moreover, we used cell surface biotinylation and confocal microscopy to demonstrate that BIN1V1 delays internalization of APP, but not of BACE1, in a RIN3-dependent pathway resulting in reduced co-localization of APP and BACE1 in early endosomes. Finally, we demonstrated that overexpression of BIN1V1 together with RIN3 dramatically reduces Aβ generation as compared to cells overexpressing BIN1V1 alone. Collectively, these findings demonstrate that neuronal BIN1 regulates trafficking, β-secretase-mediated processing of APP, and Aβ generation in a RIN3-dependent manner.

Our study began with the premise that elucidating the role of BIN1 in AD pathogenesis would require comparing the functional roles of its alternate splice variants, which show diverse tissue and cellular distribution, including in the brain. The BIN1-binding partner, RIN3, has been previously reported to translocate the BIN1 paralog, Amphiphysin II, to RAB5-positive early endosomes to initiate endocytosis^18^. Our data show that RIN3 specifically recruits the neuronal isoform of BIN1, BIN1V1, but not the non-neuronal isoform, BIN1V9, to RAB5-positive endosomes, even though both isoforms exhibited similar binding to RIN3 (Fig. 1). This is not surprising given that both isoforms contain the N-terminal SH3-domain^18^. The major difference between neuronal BIN1V1 and non-neuronal BIN1V9 is the presence of the CLAP domain in BIN1V1, and absence in BIN1V9 (Fig. 1A). A recent report has demonstrated that the CLAP-domain promotes an intramolecular interaction with the N-terminal SH3 domain of BIN1V1 to make the SH3 domain rigid, while the SH3-domain of BIN1V9 remains labile^40^. Because RIN3 binds BIN1V1 and BIN1V9 with equal affinity (Fig. 1E), RIN3-binding to the SH3-domain of BIN1V1 most likely disrupts the intramolecular interaction between the CLAP domain and the SH3 domain to “free” the CLAP-domain for interactions with the endocytic vesicles resulting in the recruitment of BIN1V1 to the early endosomes. In accord with this model, a recent study has shown that increased BIN1V1 expression inhibits propagation of tau-pathology by “sequestering” RIN3 and regulating RAB5 endocytosis^17^. Our results indicate BIN1 isoform-specific differences in the interaction of BIN1 and RIN3 in neurons versus non-neuronal cells, e.g., glia and oligodendrocytes, in the brain.

Effects of BIN1 on Aβ production have been enigmatic. While overexpression studies in SH-SY5Y and PC12 cells have shown little or no effect of BIN1 on APP-processing or Aβ generation, silencing of BIN1 in primary neurons has shown a significant increase in APP-processing and generation of Aβ (both intracellular and extracellular)^16,39,41^. Overexpression studies in PC12 cells have shown that RIN3 significantly increases β-secretase-mediated processing of APP. While it remains unclear as to whether BIN1 or RIN3 plays a direct role in APP-processing or Aβ generation, the new results presented here show that overexpression of BIN1V1 dramatically reduces β-secretase processing of APP and Aβ generation, but only when co-expressed with RIN3 (Figs. 2 and 4).

APP and BACE1 are trafficked to the cell surface prior to internalizing into early endosomes for β-cleavage and Aβ generation. Internalization of APP occurs via clathrin-mediated endocytosis and the adaptor-protein complex AP-2. In contrast, BACE1 is internalized and sorted into early endosomes via the ADP-ribosylation factor-6 (ARF6)^38^. Thus, it is intriguing that RIN3 recruits BIN1V1, which contains the CLAP (clathrin and AP2 binding)-domain, but not BIN1V9, which lacks the CLAP-domain, into the RAB5-positive early endosomes (Fig. 1). Our internalization assays showed that only cells co-expressing BIN1V1 and RIN3 delay the internalization of APP (but not of BACE1), when compared to cells expressing BIN1V1 alone (Fig. 3). These findings are consistent with reports showing that the genome-wide significant AD genetic risk-factor, CD2AP, which also binds RIN3, keeps APP and BACE1 spatially compartmentalized in neurons to regulate β-secretase processing of APP and Aβ generation^39^.

To better understand the role of neuronal BIN1V1 and RIN3 in Aβ generation, we took advantage of the unique punctate distribution of BIN1V1_GFP_ in cells co-expressing RIN3_flag_ to clearly identify the cell populations expressing BIN1V1_GFP_ + RIN3_flag_ versus cell population expressing BIN1V1_GFP_ only (Fig. 4A and Supplementary Figure S2). We found that BIN1V1 and RIN3 together, but not BIN1V1 alone, dramatically reduces the generation of Aβ (Aβ_40_ and Aβ_42_) in N2A_APP_ cells (Fig. 4B). An earlier study suggested that the lower levels of neuronal BIN1V1 observed in AD transgenic mice may “free” the steric hindrance caused by BIN1V1-RIN3 accumulation in the early endosomes to accelerate the access of APP for BACE1 and promote Aβ generation^39^. Based on our results, we conclude that RIN3-dependent exclusive recruitment of neuronal BIN1V1 in early endosomes causes steric hindrance to delay APP endocytosis resulting in spatial and temporal compartmentalization of APP and BACE1 to prevent β-secretase-mediated processing of APP and Aβ generation. Neuronal BIN1V1 is of particular interest because levels of this isoform are decreased in AD brain and have been implicated both in Aβ generation and in tau-propagation^17,39^. Other studies have also identified a strong distribution of BIN1 in microglia and oligodendrocytes^12,13^, where shorter non-neuronal isoforms may play potential roles in regulating microglial activation and myelination.

**Figure 5:**
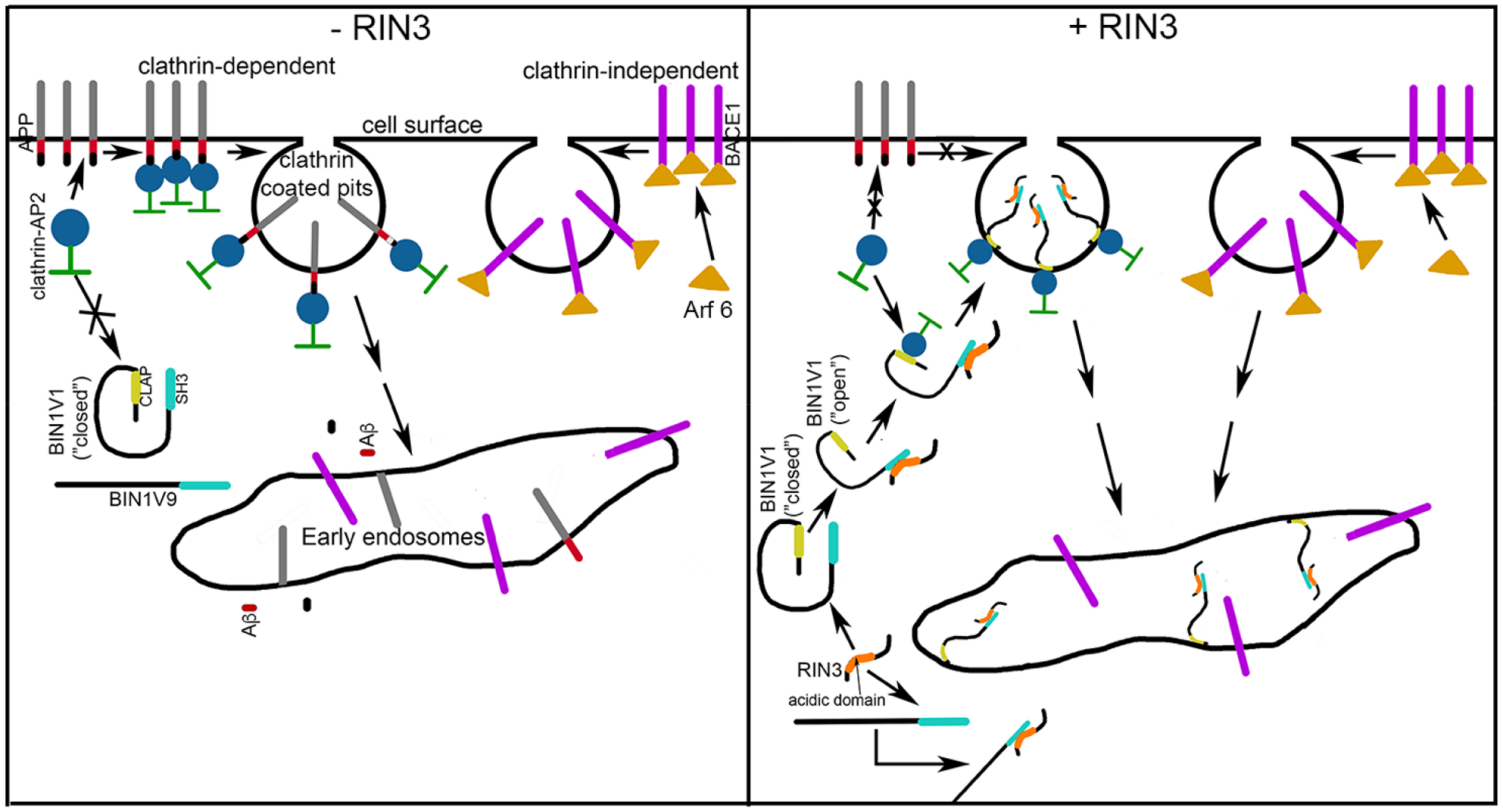
Schematic representation of BIN1V1 regulation of endosomal transport of APP in a RIN3-dependent manner. In absence of RIN3 (-RIN3, left panel) APP and BACE1 undergo clathrin-dependent (via binding to AP2-clathrin complex) and clathrin-independent (via Arf6 binding) internalization, respectively, into early endosomes where APP is cleaved by β-secretase and γ-secretase (not shown) to generate Aβ (red) and the APP intracellular domain (AICD; black). BIN1V1 remains in a “closed” form via intramolecular interaction between its CLAP and SH3 domains. (BIN1V9 does not have CLAP domain.) In the presence of RIN3 (+ RIN3, *right panel*) the SH3 domain of BIN1V1 binds to the *acidic domain* of RIN3 to “open” BIN1V1. The CLAP domain of BIN1V1 is then “open” to interact with the AP2-clathrin complex and translocates to endosomes via clathrin-dependent internalization. APP is excluded from the clathrin-pits, and is left on the cell surface, owing to either unavailability of AP2-clathrin complex or “steric hindrance” by RIN3-bound “open” BIN1V1. In contrast, clathrin-independent BACE1 internalization into early endosome remains unaltered by BIN1V1/BIN1V9-RIN3 interactions. This serves to separate APP and BACE1 into different cellular compartments, leading to attenuated generation of Aβ.

In summary, here, we assessed two genes strongly associated with AD risk, *BIN1* and *RIN3*, for their potential effects on AD pathogenesis. We showed that RIN3 differentially regulates the recruitment of neuronal BIN1V1 versus non-neuronal BIN1V9 into RAB5-endosomes, and BIN1V1, but not BIN1V9, downregulates β-secretase-mediated processing of APP. We also show that this process requires interaction with RIN3. We further showed that BIN1V1, but not BIN1V9, delays the endocytosis of APP, but not of BACE1, into early endosomes, thereby spatially and temporally compartmentalizing these two proteins, resulting in reduced β-secretase cleavage of APP and reduced Aβ generation, all in a RIN3-dependent manner (Fig. 5). These findings provide new mechanistic data regarding the molecular mechanism by which two molecules associated with AD risk, RIN3 and BIN1 (neuronal BIN1V1), interact to govern Aβ production. Collectively, these finding strongly implicate BIN1 and RIN3 as potential therapeutic targets for the prevention and treatment of AD.

## Supplementary Information

Supplementary Information.
