# Author Correction: The neuronal-specific isoform of BIN1 regulates β-secretase cleavage of APP and Aβ generation in a RIN3-dependent manner

**DOI:** 10.1038/s41598-022-09896-1

**Published:** 2022-04-06

**Authors:** Raja Bhattacharyya, Catarina Amelia Fidalgo Teves, Alexandra Long, Madison Hofert, Rudolph E. Tanzi

**Affiliations:** Genetics and Aging Research Unit, MassGeneral Institute for Neurodegenerative Disease, Henry and Allison McCance Center for Brain Health, Department of Neurology, Massachusetts General Hospital, Harvard Medical School, Boston, MA USA

Correction to: *Scientific Reports* https://doi.org/10.1038/s41598-022-07372-4, published online 03 March 2022

The original version of this Article contained an error in the spelling of the author Raja Bhattacharyya which was incorrectly given as Raja Bhattacharrya. The original Article has been corrected.

