# Supplementary material for: The neuronal-specific isoform of BIN1 regulates β-secretase cleavage of APP and Aβ generation in a RIN3-dependent manner
Source: Sci Rep. 2022 Mar 3;12:3486. doi: 10.1038/s41598-022-07372-4 (PMC8894474; doi:10.1038/s41598-022-07372-4)
Supplement: Supplementary file 1 — Supplementary Information. [file 41598_2022_7372_MOESM1_ESM.docx]

**SUPPLEMENTARY FIGURES**

Figure S1.


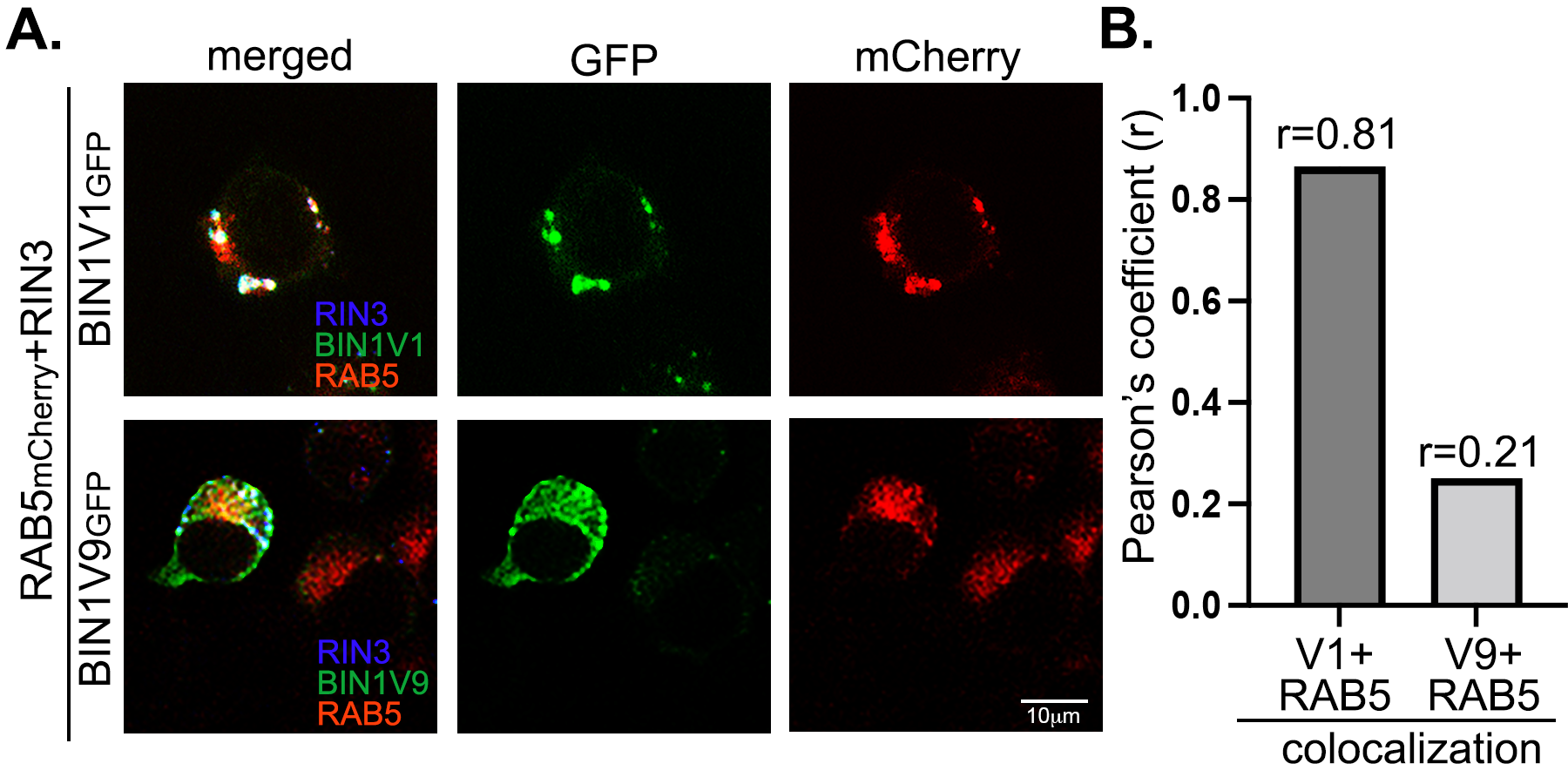


*S1. Colocalization analysis of BIN1V1/V9 with RAB5 in cells overexpressing RIN3.* **A.** Representative confocal images of cells expressing RIN3 (blue)+BIN1V1/V9_GFP_(green)+RAB5_mCherry_(red). **B.** Pearson’s coefficients (r) of BIN1V1/BIN1V9 colocalization with RAB5 in a representative single cell expressing RIN3+BIN1V1/V9_GFP_+RAB5_mCherry_. >20 cells were counted for analysis in Figure 1D.

Figure S2.


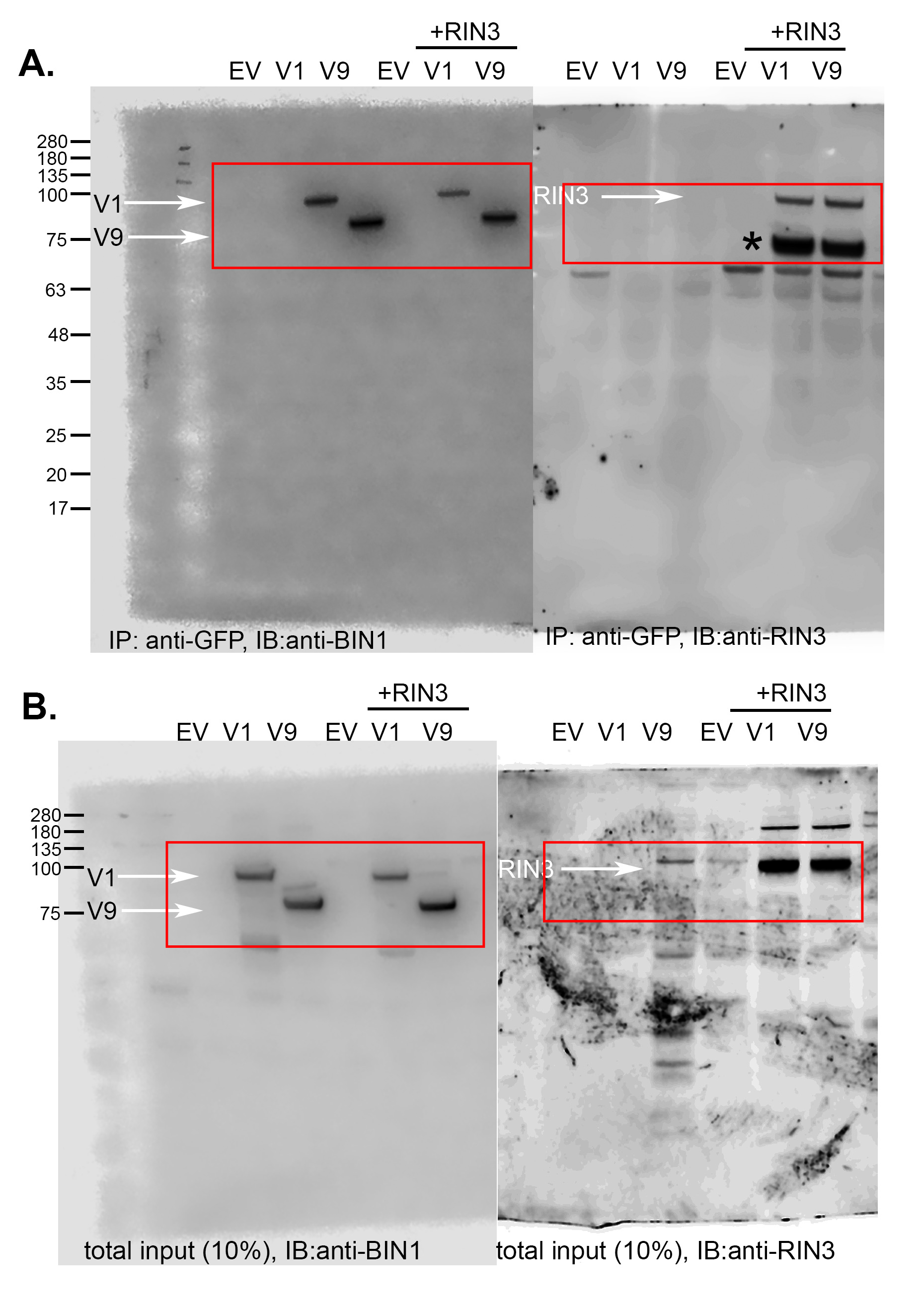


*S2. Uncropped Western blot images of BIN1V1/V9 binding to RIN3 shown in Figure 1E.* **A.** Representative Western blots of co-immunoprecipitation of cells transfected with empty vector (EV), BIN1V1_GFP_ (V1), BIN1V9_GFP_ (V9), BIN1V1_GFP_+RIN3_flag_ (V1+RIN3) or BIN1V9_GFP_+RIN3_flag_ (V9+RIN3). BIN1V1_GFP_ or BINV9_GFP_ were immunoprecipitated (IPed) with anti-GFP antibody and immunoblotted (IBed) with anti-BIN1 antibody to demonstrate pull-down of BIN1V1_GFP_ or BIN1V9_GFP_, respectively (IP: anti-GFP, IB: anti-BIN1), or with anti-RIN3 antibody to determine BIN1V1/V9 binding with RIN3 (IP: anti-GFP, IB: anti-RIN3). White arrow indicates RIN3 (~100kD). Co-IP experiments consistently detects a non-specific band at ~76kD (asterisk *). **B.** Western blot of 10% total input. Red boxes indicate the cropped images in Figure 1E.


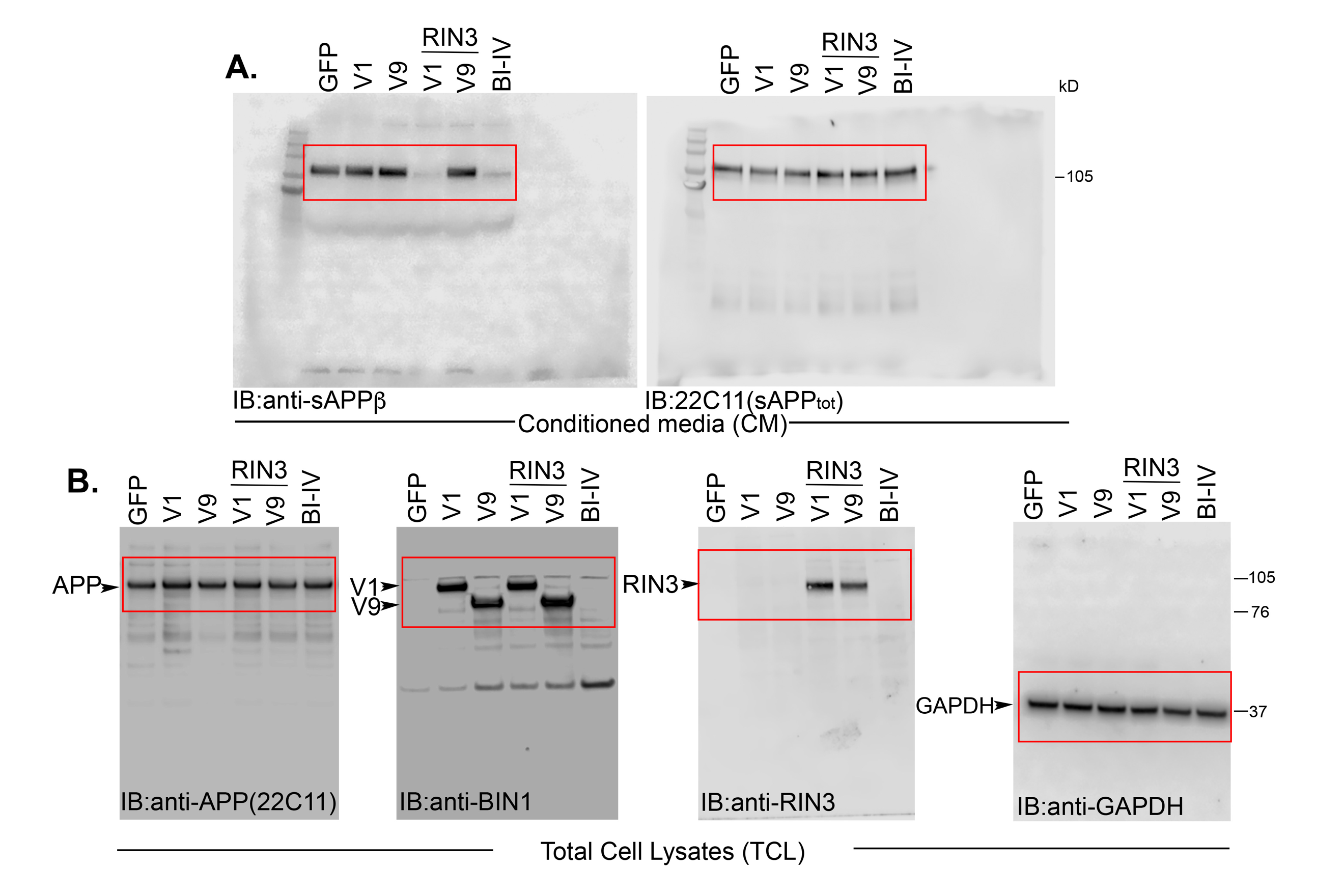
Figure S3.

*S3. Uncropped images of Western blots presented in figure 2.* **A.** Representative uncropped images of Western blots of conditioned media from N2A_APP_ cells expressing indicated proteins probed with anti-sAPPβ and anti-sAPP (22C11). B. Representative Western blot images of the total cell lysates of cells expressing indicated proteins. Anti-APP(22C11) antibody detected APP levels, anti-BIN1 antibody detected expression levels of BIN1V1_GFP_ (V1, ~100kD) and BIN1V9_GFP_ (V9, ~76kD). Anti-RIN3 detected expression the expression levels of RIN3_flag_ (~100kD). Anti-GAPDH antibody detected the levels of total GAPDH. Red boxes indicate the cropped images in Figure 2.

Figure S4.


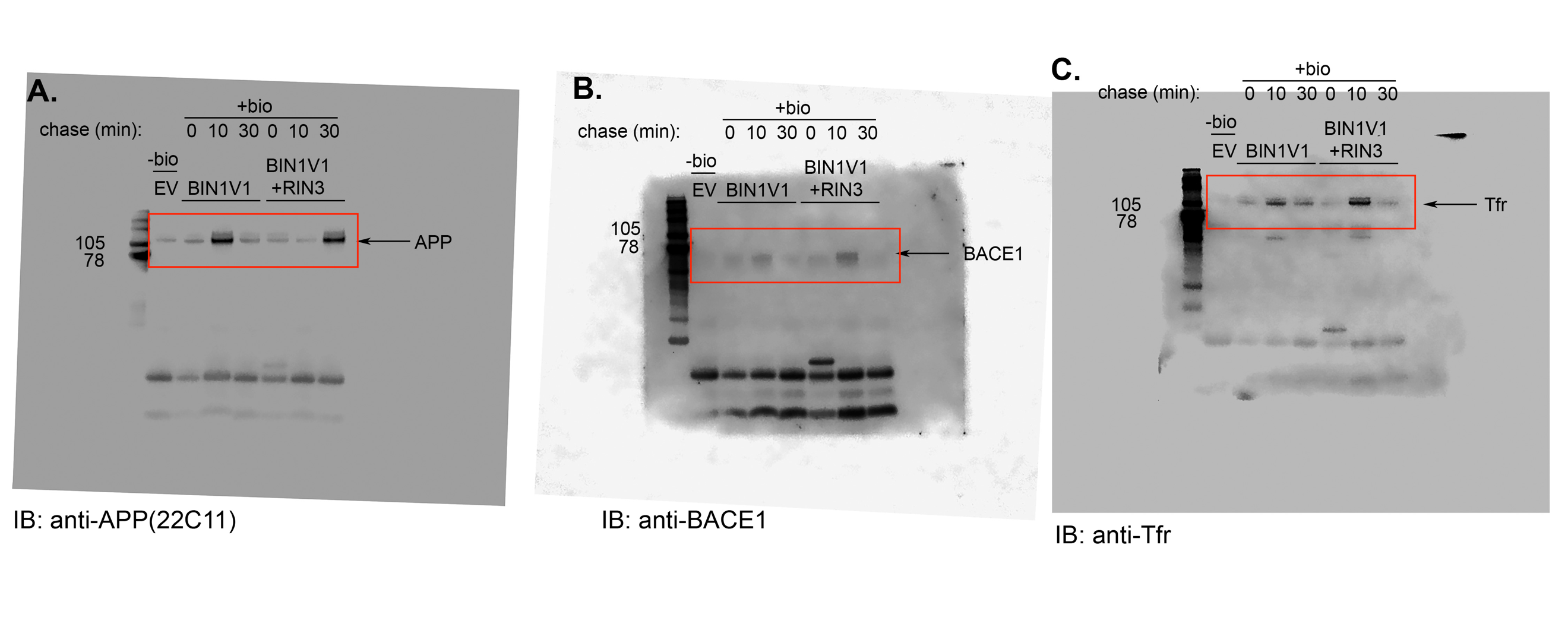
Figure S5.

*S4. Uncropped Western blots and total input of figure 3.* Representative uncropped raw images of Western blots of biotinylated internalized stably expressing APP (IB: anti-APP(22C11)) (**A.**), endogenous BACE1 (IB:anti-BACE1) (**B.**), and endogenous transferrin receptor (Tfr) (IB: anti-Tfr) (**C.**) after chasing N2A_APP_ cells for 0, 10 and 30 min at 37^O^C after arresting endocytosis for 1 h on ice. Equal amounts of proteins were loaded on neutravidin beads prior to pull-down and Western blot analyses. input. Red boxes indicate the cropped images in Figure 3.


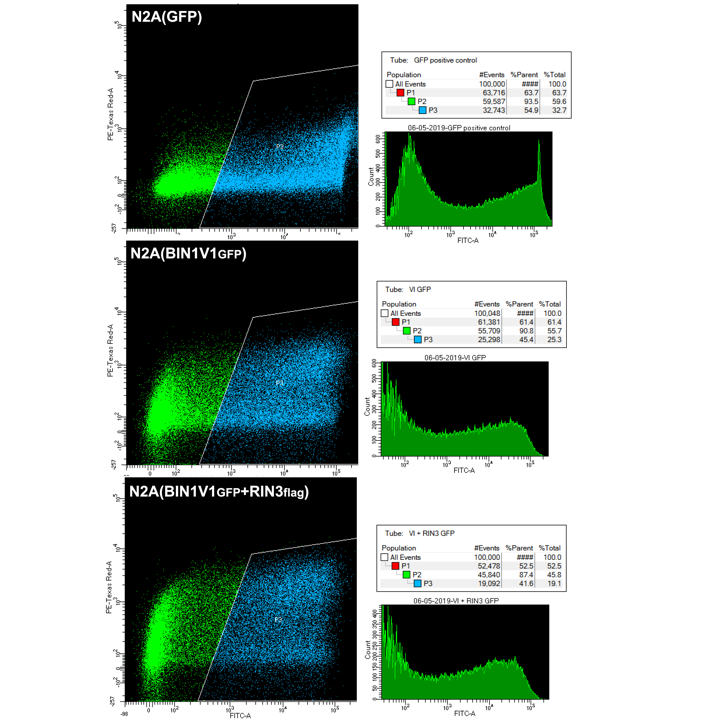


*S5. FACS analysis of cells expressing GFP and GFP-tagged proteins.* Representative FACS analysis of N2A_APP_ cells expressing GFP (N2A(GFP)), BIN1V1_GFP_ (N2A(BIN1V1_GFP_)) or BIN1V1_GFP_+RIN3_flag_ (N2A(BIN1V9_GFP_+RIN3_flag_). FACS analysis showing the number of sorted cells overexpressing GFP, BIN1V1_GFP_, and BIN1V1_GFP_+Rin3_flag_. Percent population of GFP-positive cells (P3) maximize the number of cells overexpressing GFP or the GFP-tagged proteins. The cells in P3 population were collected and grown to confluency prior to assessing the conditioned media for soluble APP or Aβ, as was done in Figures 2 and 4, respectively.
